# Supplementary material for: Unexpected Diversity of Wolbachia Associated with Bactrocera dorsalis (Diptera: Tephritidae) in Africa
Source: Insects. 2019 May 31;10(6):155. doi: 10.3390/insects10060155 (PMC6627279; doi:10.3390/insects10060155)
Supplement: Supplementary file 1 [file insects-10-00155-s001.pdf]

insects

MDPI

Article

Unexpected Diversity of *Wolbachia* Associated with *Bactrocera dorsalis* (Diptera: Tephritidae) in Africa

Joseph Gichuhi [1,2], Fathiya M. Khamis [1], Johnnie Van den Berg [2], Sunday Ekesi [1] and Jeremy K. Herren [1,3,*]

1 International Centre of Insect Physiology and Ecology (*icipe*), Kasarani, Nairobi, 00100, Kenya; jgichuhi@icipe.org (J.G.); fkhamis@icipe.org (F.M.K.); sekesi@icipe.org (S.E.)
2 Unit for Environmental Sciences and Management, North-West University, Potchefstroom, 2520, South Africa; johnnie.vandenberg@nwu.ac.za
3 MRC-University of Glasgow Centre for Virus Research, Henry Wellcome Building, Glasgow, G61 1QH, UK
* Correspondence: jherren@icipe.org

Received: 8 February 2019; Accepted: 20 May 2019; Published: date

Supplementary material

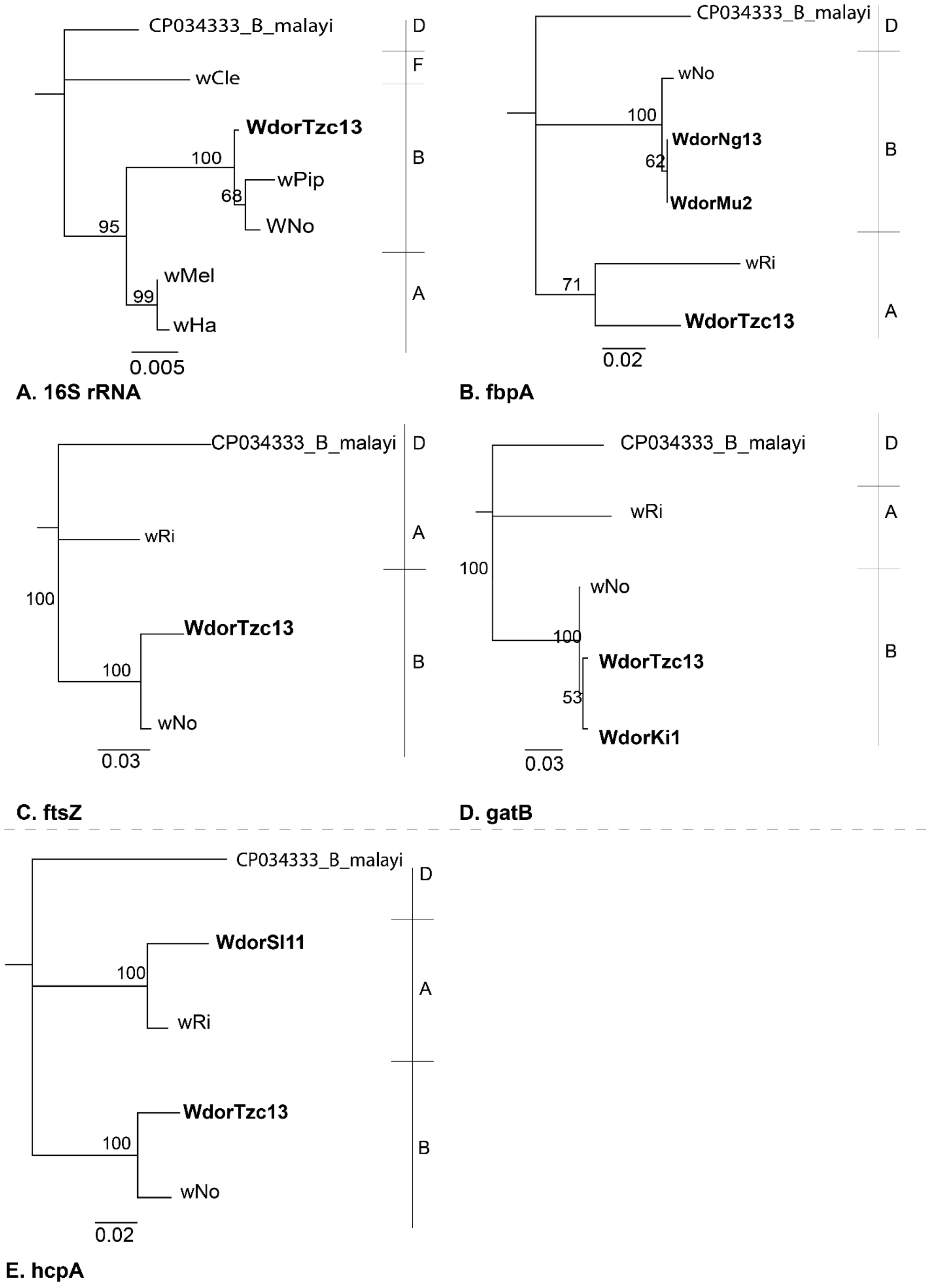

Figure 1. Neighbour joining trees showing relationship between *Wolbachia* in *B. dorsalis* (in bold) to strains in known super groups. Trees were generated from sequences of *16S rRNA* (A), *fbpA* (B), *ftsZ* (C), *gatB* (D) and *hcpA* (E) genes. Sequences of *Wolbachia* endosymbiont of *Brugia malayi* are included as out-groups in all the trees and respective super groups are indicated to the right of each tree. The detected *Wolbachia* are denoted by W followed by the host denoted as dor (*B. dorsalis*), population (Tg-Togo, Tzc-Tanzania, Ki-Kitui, Sl-Sri Lanka, Ng- Nguruman and Mu- Muranga) and population sample ID. Bootstrap values are indicated above branches. Branches with bootstrap support lower than 50% are collapsed.

Table S1. *Wolbachia* MLST and *wsp* gene allelic profiles. Exact matches to alleles present in the database are shown whereas instances where no exact matches were found are represented as N. Dashes represent the genes for each strain that could not be amplified. GenBank accession numbers for respective sequences are shown in brackets.

**Strain**	**MLST typing allele numbers**					***WSP* typing allele numbers**				
	gatB	***coxA***	***hcpA***	***ftsZ***	**fpbA**	***wsp***	**HVR1**	**HVR2**	**HVR3**	**HVR4**
WdorNg13	-	N (MK314069)	-	-	N (MK875805)	N (MK314063)	264	N	3	239
WdorMu2	-	N (MK314068)	-	-	N (MK875804)	N (MK314062)	264	N	3	239
WdorKi1	N (MK875808)	224 (MK314067)	-	-	-	N (MK314064)	N	N	N	N
WdorTzc13	N (MK875807)	73 (MK314070)	N (MK875809)	N (MK875815)	N (MK875806)	N (MK314064)	148	141	239	102
WdorSu6	-	-	-	-	-	N (MK875814)	N	N	N	N
WdorBe3	-	-	-	-	-	N (MK875811)	10	8	N	N
WdorGh4	-	-	-	-	-	N (MK875812)	N	N	N	N

WdorTg6	-	N (MK314071)	-	-	-	N (MK314065)	N	N	N	N
WdorSl6	-	-	-	-	-	N (MK875813)	N	12	21	90
WdorSl11	-	15 (MK314072)	N (MK875810)	-	-	N (MK314066)	N	8	N	N
